# Self-management education and support for type 2 diabetes in Thailand: a cluster randomized trial (2019–2021)

**DOI:** 10.1016/j.lansea.2026.100778

**Published:** 2026-05-19

**Authors:** Piyachon Aramrat, Iliatha Papachristou Nadal, Poppy Alice Carson Mallinson, Kanokporn Pinyopornpanish, Orawan Quansri, Kittipan Rerkasem, Supattra Srivanichakorn, Win Techakehakij, Nuthchanath Wichit, Kamlesh Khunti, Chaisiri Angkurawaranon, Sanjay Kinra

**Affiliations:** a Department of Family Medicine, Faculty of Medicine, Chiang Mai University, Chiang Mai, Thailand; b Department of Non-Communicable Disease Epidemiology, Faculty of Epidemiology and Population Health, London School of Hygiene and Tropical Medicine, London, UK; c Division of Care for Long Term Conditions, King's College London, London, United Kingdom; d ASEAN Institute for Health Development, Mahidol University, Salaya, Thailand; e Department of Surgery, Faculty of Medicine, Chiang Mai University, Chiang Mai, Thailand; f Research Institute for Health Sciences, Chiang Mai University, Chiang Mai, Thailand; g Royal Thai Government Ministry of Public Health, Bangkok, Thailand; h Lampang Hospital, Lampang, Thailand; i Surat Thani Rajabhat University, Surat Thani, Thailand; j Diabetes Research Centre, Department of Health Sciences, University of Leicester, Leicester, UK

**Keywords:** Diabetes mellitus, Management education, Self-care, Diabetes self-management education and support, DSMES, Primary care

## Abstract

**Background:**

Diabetes self-management education and support (DSMES) improves glycemia via improved care coordination and self-management behaviors driven by educational programs tailored to individual needs, but scalable models for its delivery in low- and middle-income countries are lacking. We evaluated the effectiveness of two scalable models of DSMES delivery: two sessions with a nurse (nurse-led) or one each with a nurse and a lay volunteer (peer-assisted).

**Methods:**

A three-arm cluster randomized trial was conducted comparing two DSMES interventions (nurse-led or peer-assisted) to routine care. The interventions consist of two DSMES sessions (once at baseline and a refresher at 6 months). The nurse-led group was delivered only by nurses, while the peer-assisted group was delivered by nurses with peer assistance. Seven primary care units were allocated to each arm. Newly diagnosed (at enrollment) or high risk glycemia (baseline HbA1c >10%) people with type 2 diabetes were eligible. Primary outcomes were differences in HbA1c and Thai-CV risk score (estimates ten-year risk of cardiovascular events in Thai population based on various clinical parameters) at 12 months relative to routine care. Secondary outcomes were individual cardiovascular risk factors.

**Findings:**

693 individuals were enrolled (mean age 59 years, 269 (39%) male, and 424 (61%) female) and 664 completed the study (4% total drop-outs). For the control, nurse-led, and peer-assisted arms respectively, the means (SD) for primary outcomes are as follows. For HbA1c: at baseline; 8.5% (1.4), 8.5% (1.9), and 8.8% (2.0), at 12-month; 7.8% (1.6), 7.7% (1.3), and 8.1% (1.6). For Thai-CV risk scores: at baseline; 22 (15), 23 (15), and 23 (14) at 12-month; 21 (14), 23 (15), and 22 (13). Mean differences at 12-month from control (95% confidence intervals) for nurse-led and peer-assisted arms respectively were: HbA1c; −0.1% (−0.4 to 0.1) and 0.1% (−0.2 to 0.5), Thai-CV risk scores; 0% (−2 to 2) and −0% (−2 to 2).

**Interpretation:**

While there were no improvements in HbA1c or CV risk scores, low drop-out rates observed demonstrate the feasibility of the models. Future explorations of the appropriate balance between the intensity and feasibility of program delivery are needed.

**Funding:**

UK 10.13039/501100000265Medical Research Council (MR/R020876/1). 10.13039/501100004396Thailand Research Fund (DBG6180007).

**Research in context**

**Evidence before this study**

Systematic reviews and randomized trials have demonstrated the effectiveness of the diabetes self-management education and support (DSMES) programs in lowering HbA1c levels and cardiovascular risk factors compared to routine diabetes care. However, such programs are not routinely available in many low-to-middle income countries (LMICs) due to constraints in the healthcare workforce available to deliver them. To identify evidence on scalable models of DSMES in LMICs we searched Pubmed without language restrictions from database inception up to July 16, 2025, using the terms (“diabetes” AND “education” AND “low and middle income”) and screened the results for randomized controlled trials of DSMES programs that reported scalable and/or low-cost models of delivering these programs (e.g. delivered by non-physician health professionals across minimal number of sessions). Our search returned no large-scale trials evaluating the effectiveness of low-cost or scalable models of DSMES delivery from LMICs.

**Added value of this study**

This study is one of the largest trials of DSMES in an LMIC. It evaluated two scalable models of DSMES delivery involving either two sessions with a nurse (nurse-led DSMES) or a single session each with a nurse and village health volunteer (peer-assisted DSMES) in northern Thailand. It found no notable differences in HbA1C and summary CVD risk scores between the interventions and control arms, suggesting that the scalable models may not be highly effective for these outcomes. Post-hoc analyses suggest potential HbA1c benefits of the scalable DSMES models in population with baseline HbA1c ≥ 10% or aged >65 years as well as improvements in fasting blood sugar and LDL-cholesterol suggesting some positive impacts on cardiovascular risk, although these findings should be considered with caution.

**Implications of all the available evidence**

Traditional DSMES programs are known to be effective but are not routinely available in LMICs due to a lack of time available to skilled healthcare professionals. This study suggests that low-cost scalable models may not be as effective but may nevertheless improve some cardiovascular risk factors. Further explorations of the appropriate balance between the intensity and feasibility of program delivery could be of value. Economic evaluations of our models and other scalable models of DSMES program will clarify the potential of such models in LMICs.

## Introduction

Globally, diabetes mellitus (DM) is among the leading causes of death and disabilities,^1^ with Type 2 diabetes mellitus (T2DM) making up over 95% of all DM cases globally.^2^ Despite the preventable and potentially reversible nature of T2DM as well as the advancements of DM treatments in recent decades, global prevalence of DM has increased over the past 30 years.^2^ Major risk factors attributed to T2DM disability-adjusted-life-years (DALYs) include; high body mass index (BMI), dietary risks factors, environmental/occupational risks, tobacco use, low physical activity, and alcohol use,^2^ which can be mitigated with life-style changes and self-management interventions.^3^, ^4^, ^5^

Diabetes self-management education and support (DSMES) is an element of diabetes care, designed to improve outcomes by giving people with T2DM the knowledge, skills, and confidence to accept responsibility for their self-management. DSMES is designed to be an ongoing care process with emphasis on collaborations between the person with T2DM and their healthcare team to help them make informed decisions, solve problems, develop personal goals and action plans, and cope with emotions and life stresses.^6^^,^^7^ DSMES program deliveries have been tested in various studies, utilizing different variations of DSMES delivery models (e.g. delivered by variations of healthcare professionals/involvement of peers/community health workers and across variations of multiple sessions and follow-up durations as well as via one-on-one/group sessions, in person and remotely). Studies in low-to-middle income countries (LMICs) have suggested that structured DSMES programs in LMICs utilizing different delivery schemes can be effective in improving glycemic control and reducing cardiovascular disease (CVD) risk.^8^, ^9^, ^10^, ^11^, ^12^ Despite the proven effectiveness from existing evidence, these studies generally included relatively intensive delivery models, either by utilizing highly trained health personnels or frequent contacts/follow-ups and sustained support systems, or both. These requirements come with significant cost and labor implications which is why structured DSMES programs are not currently routinely available in LMICs. This has led to ongoing efforts to identify more affordable and scalable DSMES delivery models for LMICs.

People with T2DM in Thailand are mostly managed in primary healthcare facilities called primary care units (PCUs). A PCU serves as an easily accessible first point of health contact located within a community, managed by a primary health care team including clinical nurses and village health volunteers (VHVs). Although they share similar roles as community health workers in many aspects, these VHVs are mostly non-professionals who volunteer to participate in the community healthcare network. They are mostly from within the local communities and therefore, have close relationships with the community. Many of the VHVs are also with T2DM or living with persons with T2DM, making them closer to peers than formal community health workers in other contexts. The rural healthcare context seen in Thailand is similar to that seen in many other LMICs with community health workers and nurses thus offering an opportunity to evaluate scalable and sustainable models of DSMES delivery that could be readily generalized to other LMICs if found to be effective.

In order to evaluate scalable DSMES delivery models, a three-arm cluster randomized controlled trial examining the effectiveness in reducing HbA1c levels and Thai CV risk scores in people with T2DM of two scalable DSMES delivery models was conducted. We hypothesized that a nurse-led DSMES and/or nurse-led DSMES with peer assistance provided by Thai VHVs (henceforth called peer-assisted DSMES) delivery models could be effective and scalable options for healthcare systems in LMICs.

## Methods

We conducted a 12-month, three-arm, parallel group, open-label cluster-randomized controlled trial, comparing two scalable DSMES delivery models (the nurse-led DSMES delivery service and the peer-assisted DSMES delivery service) to, routine diabetes management service (control). For the study clusters, any PCUs within Chiang Mai and Lampang willing to participate in the research were considered eligible for selection. The protocol for this study has already been published.^13^

Stratified by province, via computer generated randomization, we randomly selected 21 eligible PCUs to be included in the study. People with T2DM presenting to each selected cluster were then recruited from clinical records from 2019 to 2021. Recruited participants were then assessed for eligibility and asked for written informed consent before enrolment. A participant was considered eligible if they were: 1) Over 18 years of age and either was: (a) A new referral for T2DM management, or (b) A person with T2DM with high risk glycemia defined as having the latest plasma HbA1c level of >7% within the first three years of diagnosis. 2) Willing and able to attend educational group meetings. and 3) Available for 6-month and 12-month follow-up visits. Participants were excluded if they were: 1) Diagnosed with advanced diabetes complications including diabetic nephropathy, diabetic retinopathy, amputations, or if they were pregnant. 2) Diagnosed with learning disabilities, dementia, or active severe mental illness. 3) Lacking in the capacity to give voluntary, informed consent. Participant sex data were determined by birth sex stated in the citizen ID card. Data on race/ethnicity was not collected as the majority of the population covered by public providers are Thai citizens. By recruiting both those who were newly diagnosed and those with high risk glycemia who were relatively early into the disease, we intend to capture those who are relatively naïve to prior exposures of similar interventions as well as to reflect those who would likely be offered the program if adopted or widely implemented in Thailand. The rationale for exclusions was for practical challenges of participation in the program activities and reduced applicability of the program contents. After participants were screened and consent taken, we randomly allocated the 21 selected PCUs to different study arms with 1:1:1 ratio, using computer generated randomization. Due to the distinct differences in care delivery between the three arms, blinding of the interventions was not feasible, hence, we opted for an open-label design in this study. And since this was an open-label study, we opted for cluster randomization design to avoid contamination between study arms with one PCU equating to one cluster. Potential contaminations across clusters during the trial period will be assessed as a part of another process evaluation study.^14^

Participants in the routine care (control) clusters received standard diabetes care including regular clinical visits to DM clinics in the PCUs. The interval of the visits, the medication prescribed, and any health advices given were up to the considerations of the attending health providers, who were unrelated to the trial. The routine T2DM care was based on the 2017 national guideline,^15^ and also included a brief didactic educational session at the time of diagnosis of T2DM and during routine clinic visit at six months.

The two DSMES models were expected to improve disease outcomes via improvements in care coordination between health providers and patients as well as via improvements in self-management behaviors driven by educational programs tailored to individual needs. The culturally tailored DSMES delivery models (nurse-led and peer-assisted) were systematically developed using a structured process stated in the published study protocol.^13^ These DSMES deliveries were designed to utilize behavior change techniques (BCTs) such as; ‘Goals and Planning’, ‘Feedback and Monitoring’, ‘Shaping Knowledge’, and ‘Social Support’, found to be effective for non-communicable diseases (NCDs).^16^

In addition to routine diabetes care, DSMES clusters also included health education sessions consisting of four modules: Module 1. Covers the general overview of diabetes, treatment targets, and setting goals. Module 2. Covers diet and nutrition. Module 3. Covers physical activity and exercise. Module 4. Covers stress management and mental health. Each module was approximately 1.5 h and each participant was given a booklet containing information and self-assessment sections covering the contents and materials of the four modules. Seven brief videos (five to 6 min long) were developed and recorded in local language (northern Thai dialect) using local people. Designed to augment the modules, the videos were screened during the modules to standardized the contents and trigger discussions on key topic areas, covering topics such as medical adherence, dietary recommendation, physical activity, and stress management. The four modules were delivered twice as one-day sessions, once during the first month after enrolment, and another refresher session at 6 months. These sessions were conducted with groups of five to ten participants, led by the trained health professionals, and focused on engaging with the participants. In the nurse-led DSMES arm, both the first and the refresher DSMES sessions were delivered by a trained nurse. These DSMES sessions appointments were deliberately made to coincide with a regular doctor-visit day for improved accessibility and lessened resource expenditure on both the participants' as well as the providers’ sides. Participants were required to attend all sessions.

The peer-assisted DSMES arm underwent the same four modules and materials as the nurse-led arm. The first DSMES session were also led by a trained nurse but with assistance from a trained VHV, however, the DSMES refresher sessions at six months were delivered solely by the trained VHV. In addition to the DSMES sessions, participants in the peer-assisted DSMES arm also received 15-to-20-min monthly contacts from a trained VHV throughout the trial, either via house visits or telephone calls. The mode of the monthly contacts is left up to what was feasible at the time determined by each intervention site. During these brief monthly contacts, the VHV asked about the progress made regarding the self-management process, provided encouragements when needed, explored barriers to proper self-care, discussed ways to overcome the obstacles, and set new goals when necessary.

A two-day training workshop was held recruiting nurses and VHVs from the participating PCUs. The training was conducted by the research team focusing on how to use the materials and deliver the DSMES programs at the PCUs. The trial coordinators also provided periodic site visits when requested by the DSMES intervention sites throughout the trial period. These visits were meant to provide additional trainings, if needed, for the health professionals delivering the DSMES programs. A line of communication between the research team and each study site was also established across all three study arms to help with any issues that arose.

For all three study arms, the research team held weekly briefings with the study coordinators to generate a loss to follow-up participant lists. Arrangement to follow-up participants who have not turned up for their appointment were made, with attempts to contact participants through mobile text messages, phone calls, or house visits. Participants were declared lost to follow-up when they did not show up for a month and could not be contacted. Alongside the clinical trial, a process evaluation to assess the intervention delivery along with contextual factors associated with potential variations in outcomes were also assessed.^14^

Primary outcomes include differences in plasma HbA1c levels and CVD risk scores at 12 months in people with T2DM between the two DSMES arms and the control arm. CVD risk scores were calculated using the Thai-CV risk score model, which estimates the risk of dying from any CVD in Thai population over ten years based on age, gender, smoking habits, total cholesterol, and systolic blood pressure.^17^ Secondary outcomes include differences at 12 months in individual CVD risk factors, including; body mass index (BMI), waist circumference (WC), total cholesterol, low density lipoprotein cholesterol (LDL), high density lipoprotein cholesterol (HDL), triglyceride, systolic blood pressure (SBP), diastolic blood pressure (DBP), and fasting blood sugar (FBS) levels.

Blood samples and physical measurement parameters for primary and secondary outcomes were collected during a PCU visit at baseline, 6 months, and 12 months. Blood samples were taken by trained phlebotomists. Data were linked to the participant information using a unique respondent ID assigned to all study participants.

### Statistical analysis

Sample size estimation: The trial was powered to detect an absolute difference in plasma HbA1c levels of 0.6% (SD 1.5%) between control and intervention arms, based on the effect size of 0.6% noted in a previous diabetes management study in Thailand,^18^ and the fact that an increase in plasma HbA1c level of 0.5% was associated with increased mortality among people with diabetes.^19^ An intraclass correlation coefficient between PCUs of 0.02 was assumed based on similar study which found that the intraclass correlation for plasma HbA1c levels at three years was 0.02 (95% CI 0.00–0.08).^20^ When allowing for a loss-to-follow up rate of 20%, 693 participants were needed from 21 PCUs (seven in each trial arm) to achieve 80% power at 2.5% significance level. The 2.5% significance level was used to account for two primary outcomes and to favor a more conservative sample size.

Continuous demographical data are presented using mean and standard deviation values, while binary data are presented using counts and percentages within each of the study arm.

Outcome analyses: Available outcome data were analyzed on an intention-to-treat basis. Potential clustering of outcomes (HbA1c levels and Thai CV risk scores at 12 months) at the level of community PCUs were accounted for using random intercept models. Outcomes were analyzed using two multilevel regression models (accounting for province and PCU levels). For each outcome, two regression models were used to for analysis. The first model adjusted for their baseline values and the second model extended the adjustments for other relevant covariates including: age, sex, BMI, and education level. Missing outcome data were assumed missing at random. All outcome analyses were done as between-group comparisons (each intervention arm compared to routine care). Analysis outcomes were considered statistically significant at 5% significance level and the interventions are considered successful when improvements in one of the primary outcomes is observed. The statistical analyses were done using the STATA software version 15.

Combined-intervention group analysis: Since the trial was conducted during the COVID pandemic, we expected that the pandemic might have had negative impacts on both the delivery of the programs and potentially the self-managements of participants, leading to the observation of lower intervention effects. We decided to perform the combined-intervention group analysis in order to give more power to the study and potentially capture smaller effects of the DSMES programs than originally anticipated, and to capture the effects of a more generalized added support as opposed to none currently. We also performed the same analyses on all the outcomes combining all participants in the two intervention groups compared to routine care.

Sub-group analysis: Subgroup analyses of HbA1c levels by baseline HbA1C levels (HbA1c <10% and ≥10%), age (≤65 and > 65 years), gender (male and female), and level of education (<primary school and > primary school) were examined. A test for interaction was conducted to explore whether the effects of the DSMES interventions would differ by key demographics and baseline HbA1C levels. Since the CVD risk score is a composite outcome combining various parameters and only modest changes were likely to be observed in some relevant parameters, we opted out the Thai-CV risk score as additional subgroup analyses are unlikely to see any meaningful changes.

### Ethics statement

Ethical approval was obtained prior to start of the project from Chiang Mai University (No. 326/2018) and the London School of Hygiene & Tropical Medicine (16113/RR/12850). The study protocol, informed consent form, patient information sheet and other relevant information have been approved. Written Informed consent was obtained from all participants. The clinical trial was prospectively registered in April 2019, with actual patient enrollment starting in March 2020. Clinical trial number: NCT03938233.

### Role of the funding source

This study was funded by the UK Medical Research Council (MR/R020876/1) and the Thailand Research Fund (DBG6180007). The funders of the study had no role in study design, data collection, data analysis, data interpretation, or writing of the report.

## Results

**Fig. 1:**
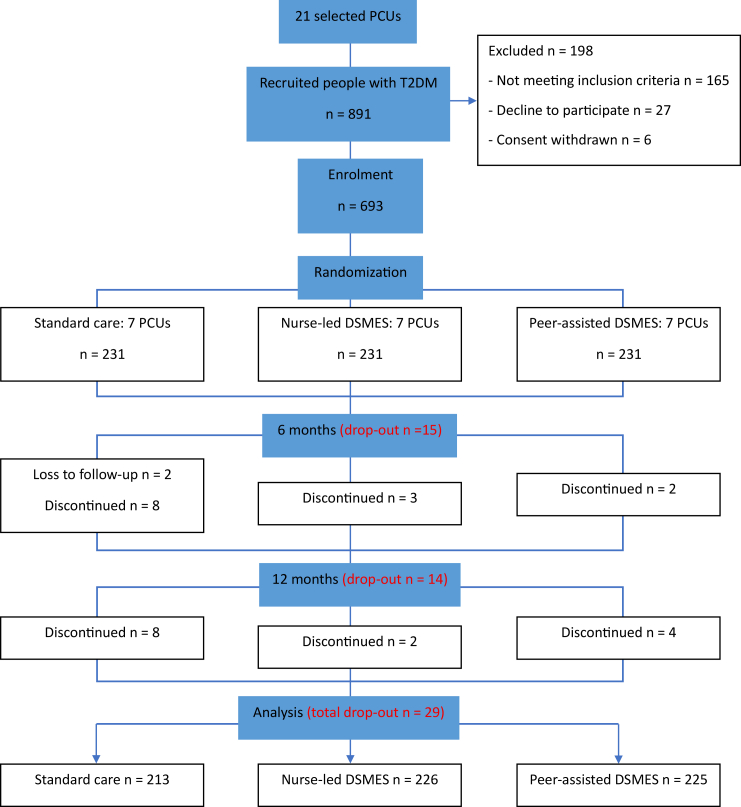
Participant flow diagram.

A total of 21 PCUs were recruited (seven PCUs for each arm). Of the 21 PCUs selected, seven were from Chiang Mai and 14 were from Lampang. The first participant was enrolled in March 2020, and last enrollment was in January 2021. Primary data collection was completed by January 2022. 891 individuals were screened for eligibility; 693 were recruited across the three arms, and 664 completed the study at 12 months (total loss to follow-up 4%), 215 from the control group, 226 for Nurse-led DSMES group, and 223 for Peer-assisted DSMES group (see Fig. 1). The distributions of the baseline characteristics between the study arms are shown in Table 1.

**Table 1:** Baseline characteristics.

Characteristics	Missing [n (%)]	Routine care (n = 231)	Nurse-led (n = 231)	Peer-assisted (n = 231)
HbA1c (%) [mean (SD)]	0 (0)	8.5 (1.4)	8.5 (1.9)	8.8 (2.0)
Thai CV risk score (%) [mean (SD)]^a^	2 (0.29)	22 (15)	23 (15)	23 (14)
Male [n (%)]	0 (0)	87 (38.20)	93 (40.26)	89 (37.99)
Female [n (%)]	0 (0)	144 (61.80)	138 (59.74)	142 (62.01)
Age (year) [mean (SD)]	0 (0)	59 (9)	59 (10)	59 (9)
BMI (kg/m^2^) [mean (SD)]	0 (0)	25 (4)	25 (5)	25 (4)
WC (cm) [mean (SD)]	3 (0.43)	88 (10)	88 (11)	84 (10)
Education level [n (%)]	2 (0.29)			
Did not study		5 (2.15)	9 (3.90)	6 (2.64)
Primary school		180 (77.25)	155 (67.10)	182 (80.18)
Junior high school		26 (11.16)	24 (10.39)	18 (7.93)
Senior high school or higher		22 (9.44)	43 (18.61)	21 (9.25)
Health insurance [n (%)]	2 (0.29)			
Universal coverage		199 (86.15)	200 (86.58)	204 (89.08)
Government official/state enterprise		12 (5.19)	11 (4.76)	10 (4.37)
Social security		20 (8.66)	20 (8.66)	15 (6.55)
Diagnosed hypertension [n (%)]	0 (0)	144 (61.80)	156 (67.53)	150 (65.50)
Diagnosed dyslipidemia [n (%)]	0 (0)	120 (51.50)	130 (56.28)	138 (60.26)
Coronary artery disease [n (%)]	0 (0)	7 (3.00)	5 (2.16)	3 (1.31)
Stroke [n (%)]	0 (0)	3 (1.29)	5 (2.16)	4 (1.75)
Drinking within 3 months [n (%)]	11 (1.59)	48 (20.96)	52 (22.91)	57 (25.22)
Smoking within 3 months [n (%)]	7 (1.01)	23 (9.96)	17 (7.46)	18 (7.93)
SBP (mmHg) [mean (SD)]	0 (0)	130 (14)	132 (15)	130 (16)
DBP (mmHg) [mean (SD)]	0 (0)	77 (9)	77 (9)	74 (10)
Triglycerides (mg/dL) [mean (SD)]	2 (0.29)	175 (110)	172 (121)	169 (139)
Total cholesterol (mg/dL) [mean (SD)]	2 (0.29)	186 (43)	190 (46)	192 (46)
LDL (mg/dL) [mean (SD)]	9 (1.30)	114 (45)	112 (43)	111 (39)
HDL (mg/dL) [mean (SD)]	1 (0.14)	48 (11)	49 (13)	50 (13)
FBS (mg/dL) [mean (SD)]	2 (0.29)	143 (36)	149 (50)	156 (52)
Total Defined Daily Dose (DDD) for diabetes medication [mean (SD)]	57 (8.23)	0.87 (0.76)	0.79 (0.82)	0.97 (0.97)
Statin use [n (%)]	57 (8.23)	109 (49.77)	108 (49.09)	118 (59.90)
Statin daily dose (mg of Simvastatin equivalent) [mean (SD)]	57 (8.23)	18.68 (8.04)	21.34 (14.24)	20.44 (10.70)
Hypertension drug use [n (%)]	57 (8.23)	144 (65.75)	136 (61.82)	109 (55.33)

a Thai CV risk score estimates ten-year risk of cardiovascular events in Thai population.

At 12 months, there was no notable differences in HbA1c and Thai-CV scores in the intervention groups compared to the control. Their respective estimated differences at 12 months, 95% confidence intervals, and p-values are shown in Table 2.

**Table 2:** Outcomes differences compared to usual care at 12 months.

	Difference (95% CI) [p-value]					
	Baseline adjustment analysis model^a^			Extended adjustment analysis model^b^		
	Nurse-led	Peer-assisted	Combined group	Nurse-led	Peer-assisted	Combined group
Primary outcomes						
HbA1c (%)	−0.1	0.1	−0.0	−0.1	0.1	−0.0
	(−0.4 to 0.1)	(−0.2 to 0.5)	(−0.3 to 0.3)	(−0.5 to 0.2)	(−0.2 to 0.5)	(−0.3 to 0.3)
	[0.46]	[0.46]	[0.95]	[0.38]	[0.44]	[0.89]
Thai CV risk score (%)^c^	0	−0	0	0	0	0
	(−2 to 2)	(−2 to 2)	(−2 to 2)	(−1 to 2)	(−2 to 2)	(−1 to 2)
	[0.81]	[0.95]	[0.92]	[0.62]	[0.88]	[0.71]
Secondary Outcomes						
BMI (kg/m^2^)	0	0	0	0	0	0
	(−0 to 1)	(−0 to 1)	(−0 to 1)	(−0 to 1)	(−0 to 1)	(−0 to 1)
	[0.29]	[0.27]	[0.22]	[0.26]	[0.30]	[0.21]
Waist circumference (cm)	2	0	1	1	−0	1
	(−0 to 4)	(−2 to 2)	(−1 to 3)	(−0 to 3)	(−2 to 2)	(−1 to 2)
	[0.07]	[0.23]	[0.22]	[0.12]	[0.95]	[0.36]
Total cholesterol (mg/dL)	1	−9	−3	1	−9	−3
	(−7 to 9)	(−17 to −0)	(−11 to 4)	(−7 to 9)	(−17 to −1)	(−11 to 4)
	[0.82]	[0.04]	[0.38]	[0.79]	[0.03]	[0.37]
LDL (mg/dL)	−9	−9	−9	−9	−9	−9
	(−18 to 0)	(−18 to 1)	(−17 to −1)	(−18 to 0)	(−18 to 0)	(−17 to −1)
	[0.06]	[0.07]	[0.03]	[0.06]	[0.07]	[0.03]
HDL (mg/dL)	3	1	2	3	1	2
	(0–5)	(−1 to 3)	(−0 to 4)	(1–5)	(−1 to 3)	(−0 to 4)
	[0.02]	[0.42]	[0.07]	[0.01]	[0.39]	[0.06]
Triglyceride (mg/dL)	7	−1	3	9	−1	4
	(−10 to 24)	(−18 to 16)	(−11 to 18)	(−8 to 26)	(−19 to 16)	(−11 to 19)
	[0.41]	[0.93]	[0.66]	[0.30]	[0.88]	[0.59]
SBP (mmHg)	−0	3	1	0	3	1
	(−3 to 3)	(−1 to 6)	(−2 to 4)	(−3 to 3)	(−1 to 6)	(−1 to 4)
	[0.98]	[0.14]	[0.43]	[0.94]	[0.12]	[0.35]
DBP (mmHg)	−1	−0	−1	−1	−1	−1
	(−3 to 1)	(−2 to 2)	(−2 to 1)	(−3 to 1)	(−2 to 1)	(−2 to 1)
	[0.51]	[0.71]	[0.55]	[0.39]	[0.59]	[0.42]
FBS (mg/dL)	−12	−7	−10	−13	−7	−10
	(−20 to −4)	(−16 to 1)	(−17 to −3)	(−21 to −5)	(−15 to 0)	(−17 to −4)
	[<0.01]	[0.07]	[<0.01]	[<0.01]	[0.06]	[<0.01]

a Multilevel regression adjusting for outcome baselines (multilevel by province and PCU).

b Multilevel regression adjusting for outcome baseline, age, sex, BMI, and education level (multilevel by province and PCU).

c Thai CV risk score estimates ten-year risk of cardiovascular events in Thai population.

Intracluster correlation coefficient (ICC) values and their respective 95% confidence intervals for primary outcomes are as follows: for HbA1c levels; 0.07 (0.03–0.12), for Thai CV risk score; 0.05 (0.02–0.13).

Moderate improvements were observed in FBS levels and some lipid parameters in each of the intervention arm and the combined-intervention analysis showed improvements in both FBS and LDL levels (see Table 2).

Examining the HbA1c outcomes by sub-groups, the post-hoc analyses suggested that there was potential effect modification by baseline HbA1c level and age (p-value for interaction <0.1) (see Table 3). Amongst patients with HbA1c >10%, the interventions showed a reduction in HbA1c when compared to usual care but was not statistically significant. Similarly, among older adults age>65, the interventions showed a reduction in HbA1c but was also not statistically significant.

**Table 3:** Differences in HbA1c at 12 months by subgroups using extended adjustment analysis model^a^ and testing for interaction with study group allocation.

Subgroups (n)	Difference (95% CI) [p-value]			Testparm for interaction with group allocation^b^
	Nurse-led	Peer-assisted	Combined group	
HbA1c ≥ 10% (121)	−0.8	−0.3	−0.5	p < 0.01
	(−1.7 to 0.2)	(−1.2 to 0.6)	(−1.4 to 0.3)	
	[0.12]	[0.50]	[0.23]	
HbA1c < 10% (540)	0.1	0.3	0.2	
	(−0.2 to 0.4)	(0.0–0.6)	(−0.1 to 0.4)	
	[0.60]	[0.04]	[0.17]	
Age >65 (145)	−0.3	−0.2	−0.2	p = 0.07
	(−0.8 to 0.3)	(−0.7 to 0.3)	(−0.7 to 0.2)	
	[0.32]	[0.41]	[0.28]	
Age ≤65 (516)	−0.1	0.3	0.1	
	(−0.4 to 0.3)	(−0.1 to 0.7)	(−0.2 to 0.4)	
	[0.74]	[0.10]	[0.49]	
Male (255)	−0.3	−0.0	−0.2	p = 0.37
	(−0.7 to 0.1)	(−0.4 to 0.4)	(−0.5 to 0.2)	
	[0.12]	[0.92]	[0.34]	
Female (406)	0.02	0.32	0.15	
	(−0.37 to 0.41)	(−0.09 to 0.73)	(−0.20 to 0.50)	
	[0.93]	[0.13]	[0.39]	
> primary school (147)	−0.1	0.1	−0.0	p = 0.99
	(−0.7 to 0.4)	(−0.5 to 0.7)	(−0.5 to 0.4)	
	[0.58]	[0.67]	[0.86]	
≤ primary school (514)	−0.2	0.1	−0.0	
	(−0.5 to 0.2)	(−0.2 to 0.5)	(−0.3 to 0.3)	
	[0.38]	[0.52]	[0.85]	

a Multilevel regression adjusting for outcome baseline, age, sex, BMI, and education level (multilevel by province and PCU).

b Three levels test for interaction with group allocation (allocation to routine care or to nurse-led DSMES or to peer-assisted DSMES).

## Discussion

In this cluster randomized controlled trial, we evaluated two distinct delivery models of DSMES program; nurse-led and peer-assisted, against routine care among individuals with recently diagnosed and people with T2DM with high risk glycemia in rural northern Thailand. The intervention did not show notable differences in the primary outcomes of HbA1c levels and Thai CV risk scores compared to routine care. Nevertheless, some secondary outcomes demonstrated notable improvements in individual cardiovascular risk factors. In particular, the combined intervention analyses indicated reductions in FBS and LDL-cholesterol levels. The post-hoc analyses suggest potential interactions between group allocation and baseline HbA1c levels and between group allocation and age. Outcome estimates from the subgroup analyses showed reductions in 12-month HbA1c in subgroups with baseline HbA1c ≥ 10% and in subgroups aged >65 years. Although the reductions observed are not statistically robust, this could be due to reduced power from substantially smaller size of comparisons. Nonetheless, cautions should be exercised when considering these results. An interesting note is that the two intervention arms seemed to showed benefits in different outcomes (the nurse-led group showed improvements in FBS and HDL, while the peer-assisted group showed improvements in total cholesterol). Only FBS and LDL seemed to be shared benefits (as found in the combined-intervention analysis). The nurse-led group delivery was assumed to be more skillful than the peer-assisted group, but the peer-assisted group was assumed to be more intensive due to inclusion of additional home visits/telephone calls. A process evaluation is currently underway to further investigate the contextual factors influencing program outcomes.^14^ No adverse events attributed to intervention activities were recorded, supporting DSMES feasibility and safety in this context.

Various existing studies have shown effectiveness of DSMES programs in LMICs.^8^, ^9^, ^10^, ^11^, ^12^ However, most of the programs with follow-up duration of at least 12 months utilized more resource intensive interventions compared to ours. These additional resources were typically utilized on either 1) health personnels (more skilled or more people), or 2) more DSMES sessions throughout the program, or both. A 2021 study in Thailand by Dermkhuntod et al. tested a DSMES model consisting of 5 DSMES visits throughout 12 months, including people with T2DM with baseline HbA1c ≥ 9%. The study focused on individuals with higher baseline HbA1c and found significant reduction in HbA1c levels of approximately 1.4%.^12^ A study in Malaysia by Ramli et al. tested a 1-year DSMES model in primary care clinics. The DSMES program were delivered by chronic disease management (CDM) team, consisted of family medicine specialists, medical officers, medical assistants/nurses, pharmacists, dieticians/nutritionists. The study found significant reduction in HbA1c levels of approximately 0.3%.^21^ Another 2019 study in China by Zheng et al. tested a DSMES model consisting of 2 DSMES sessions incorporating one-on-one nutritional guidance by dietitians and individualized exercise guidance and training by therapists and electrocardiogram monitoring. The study found significant reduction in HbA1c levels of approximately 2%.^22^

Although relatively inconsistent, some existing evidence has also shown positive effects of DSMES programs on various individual CVD risk factors such as body weight, BMI, FBS, total cholesterol, LDL, HDL, triglyceride,^10^^,^^11^^,^^23^, ^24^, ^25^ which is in line with our findings where we found clear benefits to FBS and LDL-cholesterol levels in the combined-group analysis.

A systematic review suggested that more frequent, sustained contact (>2 contacts in a month) is associated with improved DSMES outcomes.^8^ Our study's scalable approach, limiting DSMES sessions to only two sessions across 12 months, as well as the use of telephone follow-ups instead of face-to-face visits, necessitated by COVID-19 constraints during the study period, while more resource-friendly and scalable, may have diminished HbA1c benefits compared to more resource intensive DSMES programs shown to be effective in other studies. Nonetheless, although not reflected in the Thai CV risk score outcomes, benefits were seen for FBS and lipid parameters.

The potential HbA1c benefits in individuals with higher HbA1c baseline or older age suggested by our subgroup analyses could be due to: 1) individuals with higher baseline HbA1c often have higher modifiable risk factors,^26^ making them more responsive to behavioral interventions such as DSMES and 2) older adults, especially those in retirement, may also benefit more, as they often have more opportunity and motivation to engage in lifestyle changes.^27^, ^28^, ^29^ These trends support the growing consensus that “one-size-fits-all” approaches may be less effective than tailored, risk-stratified strategies.

The study's implementation highlights several important public health considerations. The low drop-out rate observed demonstrates the feasibility of adapting the DSMES programs to rural, resource-constrained environments. We believe this is achieved by leveraging readily available healthcare workers and community volunteers as well as the inclusion of local stake holders in co-designing the program deliveries. When considering implementation of the two DSMES models for general diabetic care, our study suggests that while adaptation of the two DSMES models are feasible, the lack of improvements in the primary outcomes warrant additional refinements to improve the effectiveness of the models. The appropriate balance between resource restrictions for ease of implementations versus more intense deliveries for better outcomes remains an important policy consideration. The study also suggests potentially greater glycemic benefits in higher risk groups (those with high baseline HbA1c or with older age), but the lack of empirical strengths in these findings could benefit from future additional explorations. Once more certainties are established, policymakers could consider prioritizing certain population groups which appear more likely to benefit reducing overall resource implications and open up possibilities of a more frequent/intensive DSMES delivery models found to be more effective in other studies. Finally, lessons learned regarding program delivery during disruptions such as a pandemic are valuable for ongoing and future interventions, underscoring the importance of preparedness for remote or flexible delivery models. Nevertheless, the observed improvements in individual risk factors, as well as signals from subgroup analyses, indicate that targeted, culturally tailored DSMES remains a promising strategy. Health economic evaluations focusing on these specific outcomes could further delineate the cost-effectiveness and long-term public health value.

Our study evaluates 2 DSMES sessions, utilizing only 1–2 non-physician health personnel per session. This makes our model one of the most resource-friendly DSMES delivery models studied in LMICs settings. We also tested two distinct DSMES delivery models: nurse-led and peer-assisted approaches, offering valuable options to policy makers seeking scalable interventions for low-resource settings in LMICs. The development of a culturally tailored DSMES program, designed in partnership with key stakeholders and community members, augments the program's practical relevance and acceptability. Our adaptation of short, film-based educational modules represents an innovative approach to enhance engagement, particularly among populations with low literacy, aligning with emerging evidence for multimedia health education.^30^

However, certain limitations warrant consideration. By recruiting both participants who were newly diagnosed and those with high risk glycemia to the study, we intended to reflect the intervention effects on those that were expected to be prioritized if implemented. However, the two groups may vary in motivation and response to the interventions, our study was not able to explore these relationships in detail. DSMES programs are designed to improve disease outcomes via improved self-management behaviors (such as medication usage and adherence). Since this study was not designed to capture changes in these behaviors, making inferences on how each component contributes to the changes in outcomes (or lack thereof) becomes difficult. The implementation also coincided with the COVID-19 pandemic which could potentially have negative impacts on the key components of DSMES programs in many ways and potentially attenuate the expected benefits: 1) Introducing constraints imposed by social distancing, reduced opportunities for in-person group-based activities, and shifted much of the follow-up visits to telephone-based support. 2) Potential negative impacts on health behaviors such as medical adherence due to reduced clinic visits, reduced outdoor physical activities, or diet changes due to changes in grocery patterns. 3) Potential increase in mental health burdens (e.g. increased stress, anxiety, and depression). Further explorations on these potential impacts of COVID-19 on the program implementations and outcomes will be reported as a part of another process evaluation study.^14^ Furthermore, using a composite CVD risk score to capture preventive benefits of CVDs has inherent limitations, as modest changes in individual risk factors may not translate into discernible shifts in overall calculated ten-year risk.

In conclusion, the two culturally adapted DSMES programs, delivered via nurse-led or peer-assisted models, did not lead to significant improvements in primary outcomes of HbA1c or Thai CV risk score compared to routine care. Nevertheless, there were benefits observed in individual CVD risk factors such as FBS and lipid parameters. The pragmatic, stakeholder-engaged design and successful delivery in a real-world, resource-limited setting offer actionable insights for scaling up DSMES programs. To enhance impact, future efforts should explore a more appropriate balance between intensity of delivery (e.g. increasing contact frequencies) and the ease of implementation. Extensions of follow-up time could help improve clarity on CVDs related outcomes. Health economic analyses focusing on improvements in individual CVD risk factors such as FBS and LDL will help clarify whether these delivery models are worthwhile. Our findings support the continuing adaptation and targeted implementation of DSMES as part of comprehensive diabetes and cardiovascular risk management strategies in similar settings.

## Contributors

PACM, KP, OQ, KR, SS, WT, KK, CA and SK contributed on conceptualization. IPN, PACM,KP,OW, KR, SS, WT, NW, KK, CA and SK developed the study methodology. IPN, KP, OQ, KR, SS, WT, NW, CA conducted the investigation. CA and SK supervised the study. PA, IPN, KP, CA curated the data. PACM, OQ, KR, SS,WT, NW, KK, CA, SK validated the data. PA, IPN, PACM, CA performed data analyses. IPN, KP, CA and SK administrated the project. PA, IPN, CA prepared the visualizations. PA, IPN, PACM, KP, CA and SK drafted the manuscript. All authors contributed to interpretation of the results, revised the manuscript critically for important intellectual content, and approved the final version for submission.

## Data sharing statement

None of the collected information on the individuals participated will be shared. The study protocol for this trial as well as the informed consent form used are available at https://bmjopen.bmj.com/content/10/10/e036963.

## Declaration of interests

KK has acted as a consultant, speaker or received grants for investigator-initiated studies for Abbott, Astra Zeneca, Bayer, Novo Nordisk, Sanofi-Aventis, Servier, Lilly and Merck Sharp & Dohme, Boehringer Ingelheim, Oramed Pharmaceuticals, Pfizer, Roche, Daiichi-Sankyo, Applied Therapeutics, Embecta and Nestle Health Science.
