# Supplementary material for: Self-management education and support for type 2 diabetes in Thailand: a cluster randomized trial (2019–2021)
Source: Lancet Reg Health Southeast Asia. 2026 May 19;49:100778. doi: 10.1016/j.lansea.2026.100778 (PMC13213673; doi:10.1016/j.lansea.2026.100778)
Supplement: Appendix [file mmc1.docx]

**Appendix**

1. **Summary statistics table of primary and secondary outcomes by study group allocation**

| Characteristics | Missing [n(%)] | Routine care (n=231) | Nurse-led (n=231) | Peer-assisted (n=231) |
| --- | --- | --- | --- | --- |
| Primary outcomes | | | | |
| Baseline HbA1c (% ) [mean (SD)] | 0 (0) | 8·5 (1·4) | 8·5 (1·9) | 8·8 (2·0) |
| 12-month HbA1c (%) [mean (SD)] | 30 (4·33) | 7·8 (1·6) | 7·7 (1·3) | 8·1 (1·6) |
| Baseline Thai CVD risk score (%) [mean (SD)] | 2 (0·29) | 22 (15) | 23 (15) | 23 (14) |
| 12-month Thai CVD risk score (%) [mean (SD)] | 41 (5·92) | 21 (14) | 23 (15) | 22 (13) |
| Secondary outcomes | | | | |
| Baseline BMI (kg/m2) [mean (SD)] | 0 (0) | 25 (4) | 25 (5) | 25 (4) |
| 12-month BMI (kg/m2) [mean (SD)] | 30 (4·33) | 25 (4) | 25 (5) | 25 (4) |
| Baseline WC (cm) [mean (SD)] | 3 (0·43) | 88 (10) | 88 (11) | 84 (10) |
| 12-month WC (cm) [mean (SD)] | 50 (7·22) | 87 (9) | 89 (12) | 87 (10) |
| Baseline SBP (mmHg) [mean (SD)] | 0 (0) | 130 (14) | 132 (15) | 130 (16) |
| 12-month SBP (mmHg) [mean (SD)] | 40 (5·77) | 128 (12) | 132 (16) | 131 (13) |
| Baseline DBP (mmHg) [mean (SD)] | 0 (0) | 77 (9) | 77 (9) | 74 (10) |
| 12-month DBP (mmHg) [mean (SD)] | 40 (5·77) | 76 (7) | 76 (9) | 76 (8) |
| Baseline Triglycerides (mg/dL) [mean (SD)] | 2 (0·29) | 175 (110) | 172 (121) | 169 (139) |
| 12-month Triglycerides (mg/dL) [mean (SD)] | 30 (4·33) | 166 (101) | 172 (114) | 161 (82) |
| Baseline Total cholesterol (mg/dL) [mean (SD)] | 2 (0·29) | 186 (43) | 190 (46) | 192 (46) |
| 12-month Total cholesterol (mg/dL) [mean (SD)] | 30 (4·33) | 180 (43) | 187 (42) | 176 (42) |
| Baseline LDL (mg/dL) [mean (SD)] | 9 (1·30) | 114 (45) | 112 (43) | 111 (39) |
| 12-month LDL (mg/dL) [mean (SD)] | 38 (5·48) | 113 (50) | 106 (35) | 102 (36) |
| Baseline HDL (mg/dL) [mean (SD)] | 1 (0·14) | 48 (11) | 49 (13) | 50 (13) |
| 12-month HDL (mg/dL) [mean (SD)] | 30 (4·33) | 46 (12) | 50 (14) | 49 (13) |
| Baseline FBS (mg/dL) [mean (SD)] | 2 (0·29) | 143 (36) | 149 (50) | 156 (52) |
| 12-month FBS (mg/dL) [mean (SD)] | 31 (4·47) | 148 (42) | 138 (36) | 143 (40) |

**b)Acknowledgement**

The authors would like to thank Professor Kara Hanson for her contribution during the proposal development and for her comments in preparing the manuscript.
